# Toxic Epidermal Necrolysis-Like Reaction Following Combination Therapy With Camrelizumab and Apatinib for Advanced Gallbladder Carcinoma

**DOI:** 10.3389/fonc.2021.728253

**Published:** 2021-10-29

**Authors:** Yonghao Yang, Jun Li, Brian G. Till, Jun Wang, Bicheng Zhang, Hanping Wang, Hao Huang, Tiepeng Li, Quanli Gao, Hongle Li, Zibing Wang

**Affiliations:** ^1^ Department of Immunotherapy, Affiliated Cancer Hospital of Zhengzhou University & Henan Cancer Hospital, Zhengzhou, China; ^2^ Department of Molecular Pathology, Affiliated Cancer Hospital of Zhengzhou University & Henan Cancer Hospital, Zhengzhou, China; ^3^ Clinical Research Division, Fred Hutchinson Cancer Research Center, Seattle, WA, United States; ^4^ Department of Oncology, First Affiliated Hospital of Shandong First Medical University & Shandong Provincial Qianfoshan Hospital, Shandong Lung Cancer Institute, Shandong Key Laboratory of Rheumatic Disease and Translational Medicine, Jinan, China; ^5^ Cancer Center, Renmin Hospital of Wuhan University, Wuhan, China; ^6^ Department of Pulmonary and Critical Care Medicine, Peking Union Medical College Hospital, Peking Union Medical College and Chinese Academy of Medical Sciences, Beijing, China

**Keywords:** combination immunotherapy, adverse event, PD-1, TEN, camrelizumab, apatinib

## Abstract

Recently, combination regimens based on programmed cell death-1 (PD-1) blockade have become increasingly common in clinical practice for the treatment of cancer. Such combinations significantly improve efficacy, but treatment-related adverse events have also become more complex and severe. Here, we report an acute toxic epidermal necrolysis (TEN)-like reaction in a patient with gallbladder cancer who received camrelizumab (an anti-PD-1 antibody) in combination with apatinib. Interestingly, distinct clinical and pathological characteristics were observed that differed from those of the reported cases of severe cutaneous reactions induced by anti-PD-1 antibodies alone; thus, we speculate that it was induced by the combination of camrelizumab and apatinib. It is worth noting that the TEN-like reaction showed resistance to methylprednisolone initially, which was gradually resolved after the addition of intravenous immunoglobulin (IVIg). Immunohistochemical staining revealed that the skin lesion was infiltrated by moderate numbers of CD4+ T cells and large numbers of CD8+ T cells during the progression of the TEN-like reaction, and mass cytometry by time-of-flight showed a significant reduction in the CD4+ and CD8+ T cell proportions in the peripheral blood after the rash improved. All these findings highlight the essential role of CD4+ T cells and CD8+ T cells in the TEN-like reaction induced by camrelizumab plus apatinib treatment, and we speculate that T cells, especially CD8+ T cells, attack keratinocytes. In conclusion, the TEN-like reaction induced by camrelizumab and apatinib deserves clinical attention, and further work is needed to elucidate the exact pathophysiologic mechanism as well as the optimal management strategy.

## Introduction

In recent years, immunotherapy, mostly in the form of immune checkpoint inhibitors, has progressed substantially as a treatment for different types of cancer. In particular, monoclonal antibodies targeting PD-1 provide significant clinical benefits by inducing durable regression for many types of advanced and metastatic carcinomas. However, the response rate of single-agent PD-1 blockade is only approximately 20% or lower for most advanced malignancies (1). To improve response rates, combination regimens based on PD-1 blockade are being explored, and some, such as cytotoxic T lymphocyte associated protein 4 (CTLA-4) and PD-1 dual blockades (2), chemotherapy plus PD-1 blockade (3), and anti-angiogenic tyrosine kinase inhibitors plus PD-1 blockade (4), have achieved initial success. Unfortunately, more complex and severe adverse events (AEs) often follow such combination therapies. For example, one early phase trial (5) reported that the combination of nivolumab plus pazopanib and nivolumab plus sunitinib resulted in grade 3/4 treatment-related AEs in 70% and 82% of patients, respectively, limiting future investigation of either combination regimen.

Apatinib, a selective vascular endothelial growth factor receptor-2 (VEGFR2) tyrosine kinase inhibitor (TKI), has been approved for the treatment of advanced gastric cancer in China and has also been explored in combination with an anti-PD-1 antibody, camrelizumab, with promising preliminary results against some types of solid carcinomas (6–8). Here, we reported a toxic epidermal necrolysis (TEN)-like reaction that occurred following combination treatment with camrelizumab and apatinib of a patient with metastatic gallbladder carcinoma.

## Case Description

**Figure 1:**
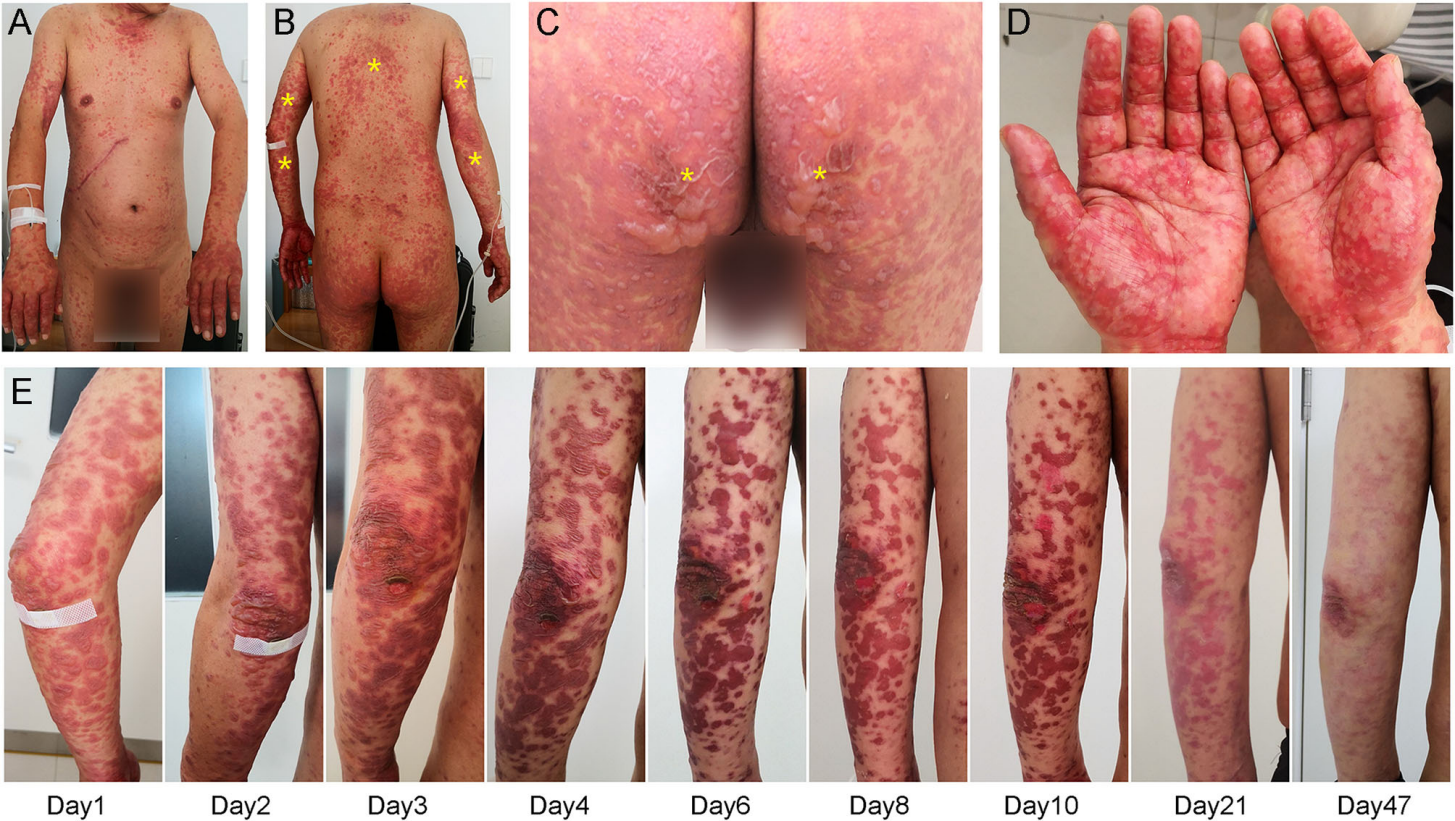
Dermatologic findings. **(A–C)** Initial inspection revealed generalized rash with blistering which was more severe in load-bearing areas (yellow asterisk), such as the hip, back, and posterior aspect of the arms. **(D)** Peripheral examination showed severe rash in the hands and feet with associated swelling. **(E)** Treatment with intravenous immunoglobulin gradually improved his rash.

A 47-year-old man with metastatic gallbladder carcinoma developed generalized pruritic maculopapular rash three days after receiving camrelizumab (200 mg, once every 3 weeks) and apatinib (250 mg, once daily) as third-line therapy. Careful review indicated no concomitant medication had been administered in the previous four weeks and that the patient had no prior history of rash. The patient discontinued apatinib treatment after consulting his doctor. Thereafter, the pruritic rash progressed rapidly, and he was admitted to our hospital five days after the combination treatment began. The clinical diagnosis of a dermatologic immune-related adverse event (irAE) was established, and he was initially treated with 80 mg methylprednisolone (1.14 mg/kg) daily for three days; however, the rash became more extensive and severe, with most of the rash becoming blisters (**Figures 1A–C**). Nikolsky’s sign was positive, and the detachable superficial epidermis comprised over 30% of the body surface area. Very interestingly, the rash was particularly severe on his hands and feet with associated swelling, which caused intolerable pain (**Figure 1D**). The rash was much more severe in areas of compression (e.g., hip, back, and posterior aspect of the arms) (**Figures 1B, C**). Simultaneously, he also felt moderate fatigue and loss of appetite. All signs indicated that the rash was rapidly worsening. After discussion with a dermatology consultant, a clinical diagnosis of TEN was established, and the severity-of-illness score for toxic epidermal necrolysis (SCORTEN) was 4, with a predicted mortality rate of 58.3%, as previously reported (9). After a skin biopsy was performed, intravenous immunoglobulin (IVIg; 30 g daily) was added to his treatment regimen, and methylprednisolone was continuously administered at the previous dose.

**Figure 2:**
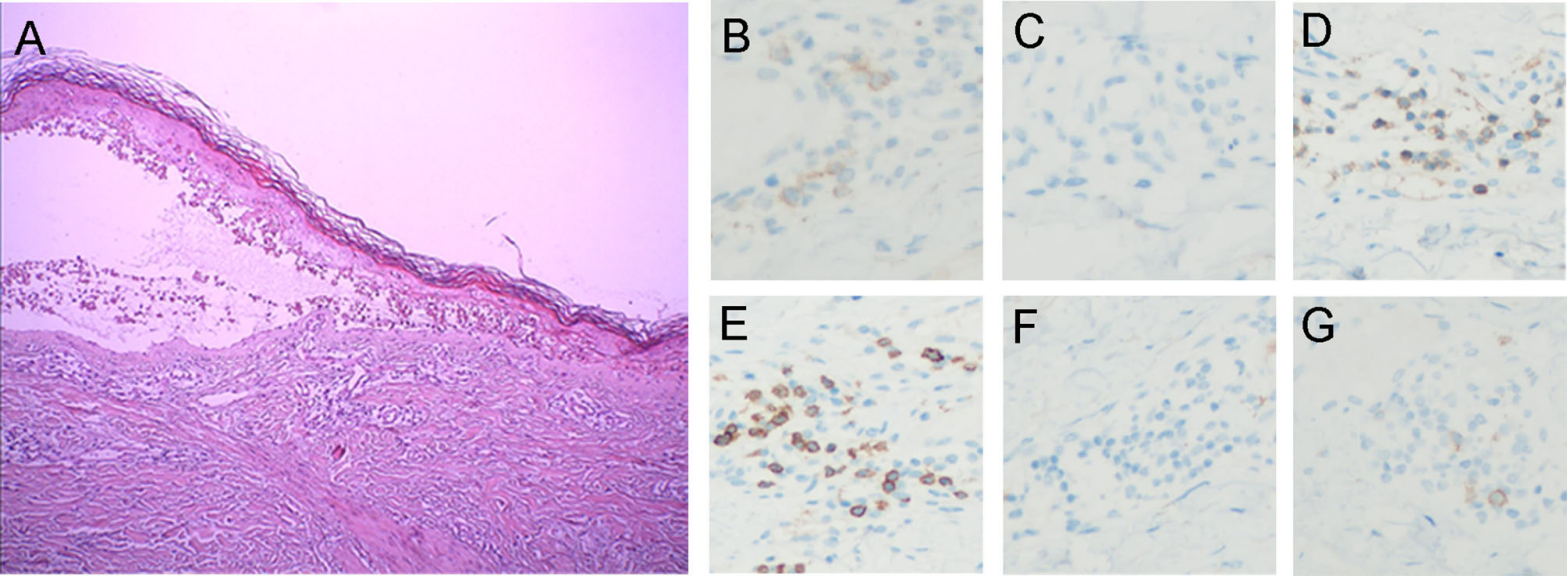
Histopathologic and immunohistochemical findings. **(A)** Histopathologic analysis demonstrated (1) epidermal hyperkeratosis with intraepidermal edema and blister formation (2), basal and suprabasal apoptotic keratinocytes with focal confluent apoptosis, and (3) moderate perivascular lymphocytic infiltration in the superficial dermis. Immunostaining shows **(B)** PD-1-positive lymphocytes (anti-PD-1, ×200) and **(C)** no PD-L1-positive cells (anti-PD-L1, ×200). Immunostaining revealed **(D)** CD4+ (anti-CD4, ×200) and **(E)** CD8+ T cell infiltration (anti-CD8, ×200). Immunohistochemistry confirmed the absence of **(F)** B cells (anti-CD20, ×200) and **(G)** NK cells (anti-CD56, ×200).

Pathologic examination (**Figure 2A**) showed epidermal hyperkeratosis with intraepidermal edema and blister formation, basal and suprabasal apoptotic keratinocytes with focal confluent apoptosis, and moderate infiltration of lymphocytes around vessels in the superficial dermis. These pathological characteristics also supported the clinical diagnosis of TEN. The day when IVIg was first administered was defined as Day 1, and since then, the rash improved daily (**Figure 1E**). IVIg was administered for five days at a cumulative dose of 150 g (> 2 g/kg), and the methylprednisolone was tapered over approximately 1 month.

More than a month after the diagnosis of TEN, the follow-up revealed complete resolution of the rash with associated exfoliation of the necrotic epidermis. However, a positron emission tomography-computed tomography (PET-CT) scan showed cancer progression compared to the pretreatment scan. After consultation, he received fourth-line therapy with albumin-bound paclitaxel. However, because of disease progression and severe lower limb neurotoxicity, this was discontinued after two cycles. Thereafter, the patient was discharged from the hospital based on his personal decision.

## Discussion

To the best of our knowledge, this is the first report of an acute severe TEN-like reaction following combination treatment with camrelizumab and apatinib. While we cannot identify the culprit drugs with certainty, we suspect that it was the drug combination. It was unlikely that the AE was caused only by apatinib, considering that no similar toxicities have been reported about apatinib, which has been widely used to treat advanced gastric cancer in China since 2014. Compared to the previously reported severe cutaneous reactions induced by anti-PD-1 antibodies (10, 11), our case has unique clinical characteristics: 1. the patient’s skin reaction occurred much earlier and progressed rapidly; 2. the patient presented with a very severe syndrome of rash, swelling, and pain in the hands and feet; 3. the rash was more severe in the load-bearing areas (e.g., hips, back, and posterior aspect of the arms) than in other areas. Potentially consistent with these findings, hand-foot syndrome is a very common adverse reaction to apatinib commonly aggravated by wearing shoes with hard soles, which could increase the pressure on the soles of the feet. Furthermore, through immunohistochemistry (IHC) analysis of the skin biopsy sample, we identified a small number of lymphocytes that were positive for PD-1 expression, and PD-L1 expression was not detected (**Figures 2B, C**). This result is not consistent with previously reported data of cutaneous adverse reactions caused by anti-PD-1 therapy alone (12), in which both PD-1 and PD-L1 expression were clearly detectable. It is well known that the interaction of PD-1 and programmed death-ligand 1 (PD-L1) is very important for immunity homeostasis (13), especially in the skin, which is the most commonly targeted organ for irAE occurrence during anti-PD-1/PD-L1 therapy (14). Therefore, we strongly suspect that the skin reaction triggered by apatinib was significantly amplified by anti-PD-1 therapy. This is also evidenced by another interesting case of severe localized well-demarcated bullous eruption which was reported in a patient with renal cell carcinoma following anti-PD-1 therapy with radiotherapy (15). The adverse reaction would not have been as severe if only radiotherapy was administered. We believe that anti-PD-1 inhibitor therapy aggravated the adverse dermatologic reaction to radiotherapy, similar to that observed in our case.

Here, we reviewed previous reports of rash associated with apatinib and/or camrelizumab in PubMed with a sample size of > 40 patients (**Table 1**). In four clinical trials about apatinib or camrelizumab monotherapy (No. 1-4) (16–19), rash was either not reported or the incidence was <10%. However, the incidence of rash observed with the combination of camrelizumab and apatinib was much higher, at over 20% or even 30%. The most convincing comparison was that of trial No. 4 (19) *versus* trials No. 6 (6) and No. 8 (21), which were all clinical trials of patients with advanced hepatocellular carcinoma in China. In these studies, the incidence of grade 1 or 2 rash observed with the combination of camrelizumab and apatinib (trials No. 6 and No. 8) was much higher than that with camrelizumab monotherapy (trial No. 4), with incidence of 28.4% and 27% *versus* <10%. Moreover, from all the studies listed in **Table 1**, the combination therapy also appeared to lead to an increased incidence of grade 3 or 4 rashes than that with apatinib or camrelizumab monotherapy. These observations suggest that combination therapy with camrelizumab and apatinib increases the frequency and severity of dermatologic AEs, consistent with the findings of our case. With the increasing use of combination therapies based on PD-1 blockade, the possibility of potentially fatal grade 3/4 dermatologic AEs will also increase, which deserves further investigation.

**Table 1:** Previous reports of rash following apatinib and/or camrelizumab therapy with sample sizes > 40 patients.

Regimen	No.	Author/year	Nature	Type of cancer	Size	Dose of apatinib	Grade 1 or 2	Grade 3 or 4
Apatinib monotherapy	1	Hu et al., 2014 (16)	Prospective	TNBC	84	750 mg or 500 mg, daily	No	No
	2	Li et al., 2016 (17)	Prospective	GC	176	850 mg, daily	No	No
Camrelizumab monotherapy	3	Huang et al., 2020 (18)	Prospective	OSCC	228	No	<10%*	<1%*
	4	Qin et al., 2020 (19)	Prospective	HCC	217	No	<10%*	<1%*
Camrelizumab plus apatinib	5	Liu et al., 2020 (20)	Prospective	TNBC	40	250 mg, daily or 7 days on/7 days off	25%	0
	6	Xu et al., 2020 (6)	Prospective	HCC	190	250 mg, daily	28.4%	0.5%
	7	Zhou et al., 2020 (7)	Prospective	Nonsquamous NSCLC	105	250 mg or 375 mg or 500 mg, daily	33.3%	1.0%
	8	Yuan et al., 2020 (21)	Retrospective	HCC	63	250 mg, daily	27.0%	3.2%
	9	Lan et al., 2020 (8)	Prospective	CC	45	250 mg, daily	26.7%	2.2%
	10	Xie et al., 2020 (22)	Prospective	Osteosarcoma	43	500 mg or 250 mg or 125 mg, daily	28.0%	4.7%

*Due to the low incidence, specific numerical values were not provided in the original article.

TNBC, triple-negative breast cancer; GC, gastric cancer; OSCC, esophageal squamous cell carcinoma; HCC, hepatocellular carcinoma; NSCLC, non-small-cell lung cancer; CC, cervical cancer.

**Figure 3:**
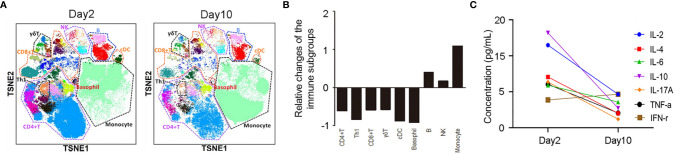
**(A)** t-distributed stochastic neighbor embedding images of immune cell subgroups in the peripheral blood of the patient on Day 2 and Day 10. **(B)** The relative log_2_-fold changes of the percentages for each immune cell subgroup reveals a significant reduction of CD4+ and CD8+ T cells with stable levels of B cells and NK cells on Day 10. **(C)** Cytometric bead array demonstrated a significant reduction of IL-2, IL-4, IL-6, IL-10, IL-17A, and TNF-α levels and slight elevation of IFN-γ levels on Day 10.

Further IHC analysis of the specimen indicated that the skin lesion was infiltrated by moderate numbers of CD4+ T cells (**Figure 2D**) and large numbers of CD8+ T cells (**Figure 2E**) but was not infiltrated by B cells (**Figure 2F**) or NK cells (**Figure 2G**). In addition, mass cytometry by time-of-flight (CyTOF) was used to compare the distribution of immune cell subgroups among peripheral blood mononuclear cells from Day 2 to Day 10. The t-distributed stochastic neighbor embedding (t-SNE) plots (**Figure 3A**) and relative log_2_-fold changes of the percentage for each subgroup (**Figure 3B**) revealed a marked reduction in the proportions of CD4+ and CD8+ T cells and stable numbers of B cells and NK cells after the rash improved on Day 10, consistent with the IHC findings. All these findings highlight the essential role of CD4+ T cells and CD8+ T cells in the progression of the TEN-like reaction induced by camrelizumab plus apatinib treatment, and we speculate that T cells, especially CD8+ T cells, attack keratinocytes, and B cells and NK cells might not have a role in this acute skin reaction. It is interesting to note that the proportion of Th1 cells within the CD4+ T cell subgroup also decreased dramatically after the rash improved (**Figures 3A, B**), suggesting the potential role of Th1 cells. This was consistent with a previous preclinical study demonstrating that PD-1 inhibition induces Th1/Th17 responses while producing fewer Th2 responses in patients with cancer (23). We also observed that the proportions of γδ T cells, conventional dendritic cells, and basophils in the peripheral blood decreased on Day 10, which also suggest a potential contribution of these immune cells to the pathogenesis of cutaneous adverse reactions. However, the exact pathophysiology of this condition remains unclear.

The diagnosis of this case was challenging. It was crucial to exclude bullous lichenoid dermatitis because it can also present with bullous lesions. Moreover, it has been reported that lichenoid dermatitis induced by immune checkpoint inhibitors (ICI) has diverse clinical manifestations, with some cases showing bullous lesions (24) However, in terms of clinical manifestations, the patient did not present with lichenoid morphology throughout the course of the illness. In terms of pathology, TEN is mainly characterized by significant epidermal necrosis, while bullous lichenoid dermatitis (including those induced by anti-PD-1 therapy) is mainly characterized by typical lichenoid/interface dermatitis (25). Although apoptotic keratinocytes can also be present in the pathology of lichenoid dermatitis, significant full-thickness epidermal necrosis does not occur. Additionally, both classic and ICI-induced lichenoid dermatitis tend to have a later onset and a longer disease course (24, 26) Therefore, lichenoid dermatitis was unlikely in the patient. Nevertheless, different from classic TEN, our case, which was likely induced by camrelizumab plus apatinib, did not present with mucosal involvement and prodromal fever, and the distribution of his rash was unique, so the patient was diagnosed with a TEN-like reaction.

The treatment of this case is also worth discussing. In classic TEN, many cytotoxic T lymphocytes (CTL) associated cytokines, chemokines, or cytotoxic proteins are involved in epidermal necrolysis, especially granulysin (27) and TNF-α (28), and the interaction between Fas and Fas ligand (FasL) also participates in the process but is not the main pathomechanism. To this patient with a TEN-like reaction and glucocorticoid resistance, IVIg was administered; subsequently, surprisingly good results were achieved. Based on the previous studies on classic TEN (29), one possible mechanism is that IVIg contains anti-Fas antibodies, which could block the FasL-mediated apoptosis of keratinocytes. Therefore, we hypothesized that the severe skin reaction induced by apatinib and camrelizumab has a pathological mechanism that is not completely consistent with the classic TEN, and perhaps the interaction between Fas-FasL plays a key role. In a line with this, we speculated the T cells, especially CD8+ T cells (CTL), attack keratinocytes, and as is known, Fas-FasL is an major pathway for both CD4+ T cell- (30) and CD8+ T cell- mediated (31) cytotoxicity.

The contribution of methylprednisolone to the improvement of the rash should not be overlooked either. In our case, cytometric bead array (CBA) was used to compare the concentrations of seven cytokines (IL-2, IL-4, IL-6, IL-10, IL-17A, TNF-α, and IFN-γ) in the peripheral blood between **Day 2** and Day 10. On Day 10, the levels of all cytokines except IFN-γ significantly decreased (**Figure 3C**), concomitant with the improvement of rash. These findings might be attributed to the use of methylprednisolone, as a previous study demonstrated that methylprednisolone could decrease the peripheral cytokine levels (i.e., IL-4, IL-6, IL-10, IL-17, IFN-γ, and TNF-α) in patients with rheumatoid arthritis (32). Moreover, it might be possible that IFN-γ levels on **Day 2** had decreased significantly because during that time, methylprednisolone had already been used for four days.

Another topic worth discussing is the discontinuation of the anti-PD-1 therapy. Undoubtedly, continuing anti-PD-1 therapy may re-induce severe skin reactions, but on the other hand, emerging data suggest that the development of cutaneous toxicity may correlate with effective response to ICI therapy in patients with metastatic melanoma (33, 34). In fact, cutaneous irAEs could be a surrogate for clinical benefit. Therefore, it would be important that the ICI therapy is continued despite cutaneous toxicities, particularly because they can be managed without the discontinuation of therapy. The PET-CT scan showed disease progression; however, the continuation of ICI therapy might have resulted in the stabilization of the disease, and even reduction of the tumor burden. In fact, delayed clinical responses, such as an increase in total tumor burden (pseudoprogression) followed by tumor regression have often been observed in studies of immunotherapeutic agents since these agents may require additional time to achieve measurable or sustained clinical effects compared to traditional cytotoxic chemotherapy (35).

Currently, a variety of PD-1-based combination regimens have emerged, most of which demonstrated better clinical efficacy, but were always accompanied with increased toxicities. Consistent with this, a meta-analysis of nivolumab plus ipilimumab combination therapy raised the issue that the deleterious effects of severe irAEs might outweigh the benefit from the addition of ipilimumab (36). Recently, however, Kleef et al. demonstrated that the use of off-label low doses of nivolumab and ipilimumab in a combined treatment resulted in irAEs that were significantly safer than those observed upon following the established protocols without compromising efficacy (37). This may also give us a clue: it is important to design a rational combination regimen to reduce adverse events without compromising efficacy, or with even improving the anti-tumor effect.

In conclusion, we reported the first acute TEN-like reaction likely induced by the combination of camrelizumab and apatinib, which showed resistance to methylprednisolone but was gradually resolved after the addition of IVIg. According to the pathological analysis and examination of the changes of the immune cell subgroups in the peripheral blood, we speculate that T cells, especially CD8+ T cells, attack keratinocytes. Further work is needed to elucidate the exact pathophysiologic mechanism as well as the optimal management strategy. 

## Patient Perspective

Written informed consent for the publication was obtained from the patient. From patient perspective, he was not satisfied with the combination therapy of apatinib and camrelizumab, not only because the tumor was not under control, but also because of the fear of the rash. However, the patient was very satisfied with the treatment of the rash. Although the rash developed rapidly during the initial phase of methylprednisolone therapy and caused great anxiety and fear, it gradually improved after the addition of IVIg, without significant treatment-related adverse reactions.

## Data Availability Statement

The original contributions presented in the study are included in the article/supplementary material. Further inquiries can be directed to the corresponding authors.

## Ethics Statement

The studies involving human participants were reviewed and approved by Henan Cancer Hospital Medical Ethics Committee. The patients/participants provided their written informed consent to participate in this study. Written informed consent was obtained from the individual(s) for the publication of any potentially identifiable images or data included in this article.

## Author Contributions

YY and JL collected data and wrote the manuscript. ZW conceived and corrected the manuscript. BT reviewed and edited the manuscript. HH and TL performed the immunostaining and cytokine detection work, respectively. All authors commented on and corrected the manuscript. All authors contributed to the article and approved the submitted version.

## Funding

This work was supported by the Henan Medical Science and Technology Research Plan (Grant No. LHGJ20190646), the National Natural Science Foundation of China (Grant No. 81972690, 81000914, and 81272526), the Medical Science and Technology Research Project of Health Commission of Henan Province (2018010033) and the Henan Provincial Scientific and Technological Project (212102310750). The funding bodies played no role in the design of the study; the collection, analysis, and interpretation of data; or manuscript preparation.

## Conflict of Interest

The authors declare that the research was conducted in the absence of any commercial or financial relationships that could be construed as a potential conflict of interest.

## Publisher’s Note

All claims expressed in this article are solely those of the authors and do not necessarily represent those of their affiliated organizations, or those of the publisher, the editors and the reviewers. Any product that may be evaluated in this article, or claim that may be made by its manufacturer, is not guaranteed or endorsed by the publisher.

## Abbreviations

AEs, Adverse events; CBA, Cytometric bead array; CTL, Cytotoxic T lymphocytes; CTLA-4, Cytotoxic T lymphocyte associated protein 4; CyTOF, Mass cytometry by time-of-flight; ICI, Immune checkpoint inhibitors; irAE, Immune-related adverse event; IHC, Immunohistochemistry; PBMC, Peripheral blood mononuclear cells; PET-CT, Positron emission tomography-computed tomography; PD-1, Programmed cell death-1; SCORTEN, Severity-of-illness score for toxic epidermal necrolysis; t-SNE, t-distributed stochastic neighbor embedding; TEN, Toxic epidermal necrolysis; TKI, Tyrosine kinase inhibitor; VEGFR2, Vascular endothelial growth factor receptor-2.
